# Supramolecular Association between γ-Cyclodextrin and Preyssler-Type Polyoxotungstate

**DOI:** 10.3390/molecules26175126

**Published:** 2021-08-24

**Authors:** Nathalie Leclerc, Mohamed Haouas, Clément Falaise, Serge Al Bacha, Loïc Assaud, Emmanuel Cadot

**Affiliations:** 1 Institut Lavoisier de Versailles, UMR 8180 CNRS, UVSQ, Université Paris-Saclay, 78035 Versailles, France; clement.falaise@uvsq.fr (C.F.); serge.albacha@universite-paris-saclay.fr (S.A.B.); emmanuel.cadot@uvsq.fr (E.C.); 2 Institut de Chimie Moléculaire et des Matériaux d’Orsay (ICMMO)—ERIEE, UMR 8182 CNRS, Université Paris-Saclay, 91400 Orsay, France; loic.assaud@universite-paris-saclay.fr

**Keywords:** polyoxometalate, supramolecular adduct, XRD structure, NMR spectroscopy, cyclic voltammetry, electrochemical impedance spectroscopy

## Abstract

The development of hybrid materials based on polyoxometalates constitutes a strategy for the design of multifunctional materials. The slow evaporation of an aqueous solution of [NaP_5_W_30_O_110_]^14−^ in the presence of γ-Cyclodextrin (γ-CD) led to the crystallization of a K_6_Na_8_{[NaP_5_W_30_O_110_]•(C_48_H_80_O_40_)}•23H_2_O (**NaP_5_W_30_•1CD**) supramolecular compound, which was characterized by single-crystal X-ray diffraction, IR-spectroscopy, thermogravimetric and elemental analyses. Structural analysis revealed the formation of 1:1 {[NaP_5_W_30_O_110_]•[γ-CD]}^14−^ adduct in the solid state. Studies in solution by cyclic voltammetry, electrochemical impedance spectroscopy, ^1^H NMR spectroscopy, and ^31^P DOSY, have demonstrated weak interactions between the inorganic anion and the macrocyclic organic molecule.

## 1. Introduction

Polyoxometalates (POMs) correspond to water soluble metal-oxide discrete polyanions based mostly on Mo or W in their highest oxidation states (M^VI^) [1]. POMs can include in their metal-oxo framework almost all the elements of the periodic table, featuring various electronic configurations and bonding patterns, which impart an unmatched and tunable range of physical and chemical properties [2]. Therefore, this class of compounds has been intensively studied and used in a wide range of applications such as catalysis [3], nanoscience [4], medicine [5], and energy conversion and storage [6]. However, most of these applications require the integration of POMs within devices, bulk matrices, or interfaces, as close as possible to target molecules or functional groups. Different strategies were employed for integrating POMs into functional systems, such as the covalent attachment [7], electrostatic pairing [8], or supramolecular attraction [9]. 

**Figure 1:**
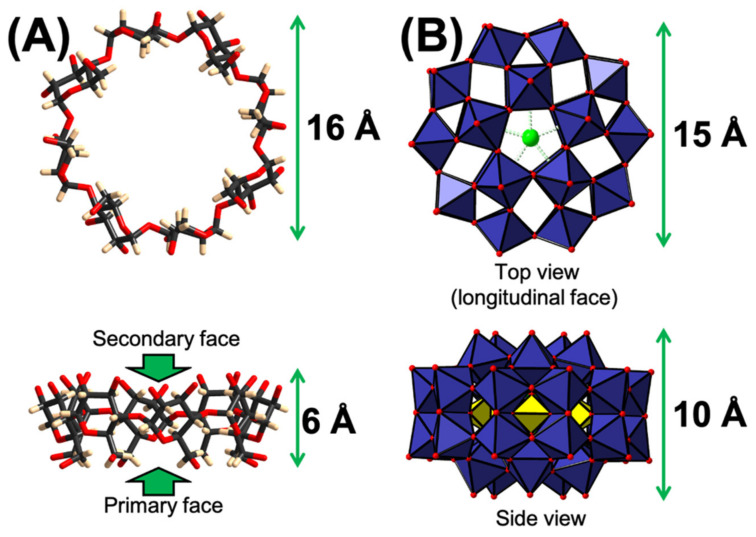
(**A**) Structural representations of the molecular entities used as building blocks. (**A**) γ-cyclodextrin C_48_H_80_O_40_ (γ-CD) resulting from the condensation of eight glycopyranose units. The toroidal macrocycle presents two distinct faces, a smaller one delimited by 8 methoxy groups (primary) and a wider one (secondary) delimited by 16 hydroxyl groups. (**B**) The Preyssler anion [NaP_5_W_30_O_110_]^14−^ with oblate spheroid shape presents two main (longitudinal) faces perpendicular to the virtual *C*_5_ symmetry axis.

One of the most striking supramolecular properties of POM anions arises from their ability to form in aqueous solution inclusion complexes with toroidal cyclodextrin macrocycles (CDs) [10]. These oligosaccharides made of 6 (α), 7 (β) or 8 (γ) glucopyranose units exhibit a hydrophobic internal cavity capable of hosting a large variety of inorganic compounds of appropriate size and shape [11]. These macrocyclic cavitands have found utility in various fields of application such as catalysis [12], materials science [13], sensing [14], and drug delivery [15]. Driven by the chaotropic effect, CDs can remarkably encapsulate either cationic [16], anionic [17,18,19,20] or even neutral metallic species [21]. This effect consists of a water structure recovery process resulting from the desolvation of both the chaotropic species and the host molecule [17,22]. The chaotropic character of POMs has now been definitively recognized, and some of them have even been identified as superchaotropic entities according to the Hofmeister classification [23,24]. The strength of these supramolecular host−guest associations can vary considerably depending on the shape, size and most importantly the charge density of the POM [23,25]. Among the large family of POM structures, known examples of complexation with CD include Lindqvist [26], Keggin [23,27], and Dawson-type POMs [22]. In addition, inclusion complexes with the gigantic blue wheel [Mo_154_O_462_H_14_(H_2_O)_70_]^14−^ have also been observed with role reversal where the cyclic POM acts as a CD-encapsulating host [28]. In this contribution, we report the synthesis, crystal structure, solution behavior and electrochemical properties of a new supramolecular adduct between γ-CD and another POM-type, namely the Preyssler anion [NaP_5_W_30_O_110_]^14−^. This POM anion exhibits an oblate ellipsoid shape with a size comparable to that of γ-CD (Figure 1), where classic host−guest inclusion complexes are not expected. This study would provide an original approach to reach new hybrid POM–CD composites.

## 2. Results and Discussion

**Figure 2:**
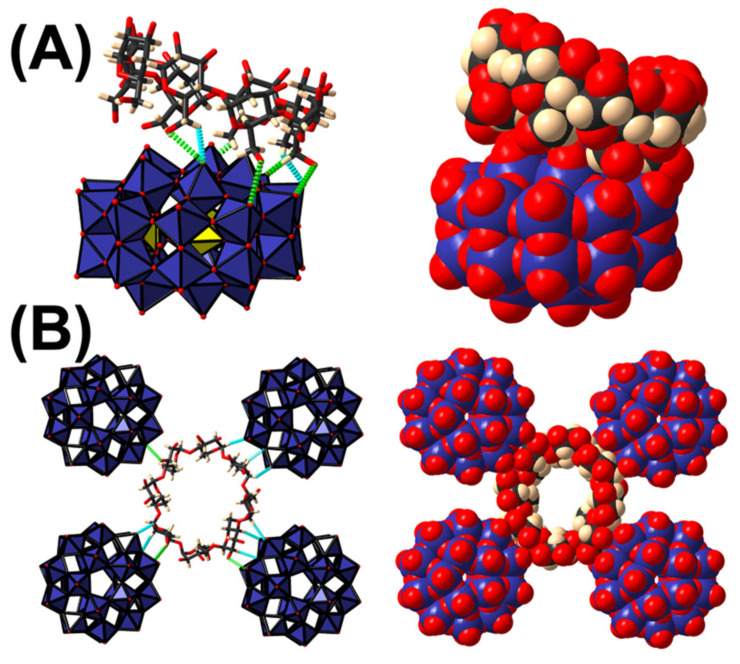
(**A**) Mixed representation of the supramolecular assembly observed in the solid-state involving γ-CD and the Preyssler anion [NaP_5_W_30_O_110_]^14−^. (**B**) Mixed representation of the hybrid layer resulting from the interaction between the external surface of the organic macrocycle and the polyoxoanions. Hydrogen bonds (O-H•••O-W) and short contacts (H•••O-W) are represented in dashed green and blue lines, respectively.

Single crystals of **NaP_5_W_30_•1CD** were grown from the slow evaporation of an aqueous solution containing [NaP_5_W_30_O_110_]^14-^ anion, γ-CD and sodium chloride (see experimental details Section 3.5). The combination of different complementary techniques (elemental analysis, XRD and TGA) had led to the molar composition of **NaP_5_W_30_•1CD** as K_6_Na_8_{[NaP_5_W_30_O_110_]•1(C_48_H_80_O_40_)}•23H_2_O. Single crystal X-ray diffraction analysis revealed that **NaP_5_W_30_•1CD** is built from face-to-face supramolecular 1:1 hybrid adduct {[NaP_5_W_30_O_110_]•[γ-CD]}^14−^ (see Figure 2A). The geometrical parameters of the Preyssler ion range within those previously observed structures [29,30]. The pocket inside the anion is filled by one sodium cation delocalized on two positions with a 0.5 occupancy factor. In this position, the inner Na^+^ ion is bound to five oxygen atoms P-O-W of the polyanion with bond lengths in the range 2.6 ± 0.1 Å. Within the supramolecular arrangement {[NaP_5_W_30_O_110_]•[γ-CD]}^14−^, the γ-CD exposes its primary face to the longitudinal face of the inorganic crown unit. As generally observed for POM/cyclodextrin assemblies, the two components are held together by a set of weak attractive interactions including hydrogen bonds and short contacts. Seven methoxy units (over eight) of the primary face interact with the POM surface through hydrogen bonds between hydroxo groups and the terminal oxygen atoms of POMs (d_O•••O=W_ = 2.9 ± 0.1 Å) and a few short contacts involving H6 protons of the CD and terminal and bridging oxygen atoms of the POM surface (d_H6•••O-W_ = 2.7 ± 0.2 Å). Usually inclusion-like and sandwich-type complexes are observed with smaller POMs such as Keggin or Dawson-type structures [22,23]. With the large anionic Preyssler-type polyanion, such arrangements are not possible, highlighting the importance of the host−guest size matching condition for inclusion complexation. The external surface of the tori host interacts also with four adjacent POM units through hydrogen bonds and short contacts forming a pseudo-square arrangement in which the center is occupied by the CD. Such an organization is probably favored by the local symmetry of the organic macrocycle (pseudo *C*_8_). The two types of supramolecular association between the POM and the γ-CD (primary face and external surface) lead to an extended framework with γ-CD forming double layers of interconnected [NaP_5_W_30_O_110_]^14-^ and CD motifs through bridging alkali cations (see Figure S4 in the Supplementary Materials). Indeed, most of cations (5 K and 6 Na) have been localized within the structure. They are involved in electrostatic interactions with both the POM and the CD considering the short contacts in both O•••K•••O and O•••Na•••O junctions between terminal {W=O} groups (O•••K or O•••Na distances ~2.8 ± 0.1 Å) and oxygen atoms of the glucopyranose moieties. 

To investigate the interaction between the γ-CD and the Preyssler-type anion in solution, titration experiments were conducted by means of complementary electrochemical techniques, namely cyclic voltammetry (CV) and electrochemical impedance spectroscopy (EIS), and ^1^H nuclear magnetic resonance (NMR) spectroscopy. In addition, ^31^P DOSY NMR was also employed on some selected samples.

**Figure 3:**
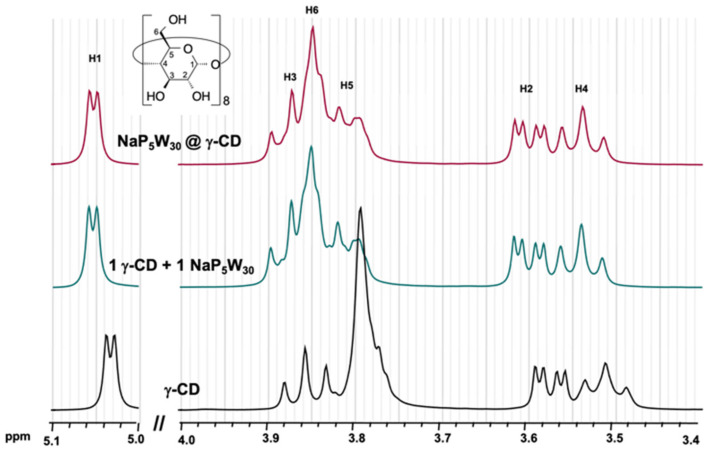
^1^H NMR spectra of aqueous solution 10 mM γ-CD, an equimolar solution (10 mM) of a mixture of K_14_[NaP_5_W_30_O_110_] and γ-CD, and 10 mM aqueous solution of **NaP_5_W_30_•1CD** complex.

^1^H NMR spectroscopy in D_2_O is the technique of choice to study the behavior of cyclodextrin in solution since the chemical shift is highly sensitive to the formation of complexes. A study of titration of aqueous γ-CD solution with the Preyssler anion shown in Figure S5 revealed among the six proton types of the molecule, only H6 of the methoxy hanging groups underwent significant low-field shift. Although modest (Δδ = 0.05 ppm), this effect is fully consistent with the solid-state structure of the supramolecular adduct, showing close contact between the POM and the primary face of the CD. Modeling the chemical shift variation provides an estimation of association constant *K* of 430 ± 60 M^−1^ (see Figure S6). To further evidence the supramolecular interaction maintained in solution, the ^1^H NMR spectrum of the isolated compound **NaP_5_W_30_•1CD** was compared to the spectrum of an equimolar solution of [NaP_5_W_30_O_110_]^14−^ and γ-CD (Figure 3). The spectra were almost identical, both showing a significant shift towards the low-field of the H6 resonance as already observed in the titration experiment. We demonstrated recently that such an interaction in solution is governed by the chaotropic character of the POM that depends mainly on its charge density [23]. By comparing these results with those previously reported for Keggin-type POMs, the modest variations in NMR shifts observed for the Preyssler anion are reminiscent of the solution behavior of the [H_2_W_12_O_40_]^6−^ ion, both exhibiting a similar charge density (charge *q* per W atom = 0.5) [23].

The ^31^P NMR was measured in an attempt to probe these supramolecular interactions from the POM side. No difference in chemical shifts (−9.1 ppm) was observed between the spectrum of the 10 mM solution of compound **NaP_5_W_30_•1CD** and the spectrum of a 10 mM reference solution of [NaP_5_W_30_O_110_]^14−^ (Figure S7). However, the DOSY ^31^P NMR revealed a slight decrease in the self-diffusion *D* of the Preyssler anion from 213 to 193 µm^2^/s between the two solutions indicating that presence of one γ-CD per POM slows down the mobility of the anion in solution.

**Figure 4:**
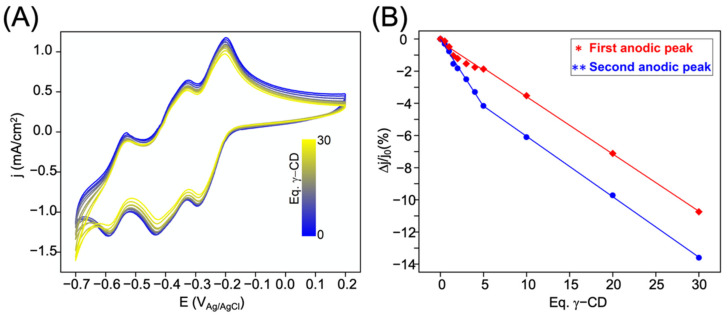
(**A**) Cyclic voltammograms of the [NaP_5_W_30_O_110_]^14−^ anion (1 mmol·L^−1^, glassy carbon working electrode, scan rate 200 mV·s^−1^) in the presence of increasing amounts of γ-CD (from 0 to 30 equivalents). The experiments have been performed in 50:50 (*v:v*) HCl (0.1 mol·L^−1^):NaCl (0.9 mol·L^−1^) aqueous solution (pH = 1.3). (**B**) Plot of the variation of the first (red filled diamond-shaped symbol) and the second (blue filled circle symbol) cathodic peaks extrema versus equivalent number of γ-CD showing a break point at ca. 5 eq. CD (j_0_ = cathodic peak current density of [NaP_5_W_30_O_110_]^14−^ anion without γ-CD).

The complexation of the [NaP_5_W_30_O_110_]^14−^ anion by γ-CD can also be monitored through the change of the electrochemical response of the POM in the presence of an increasing amount of the CD. The resulting CVs obtained in the presence of various amounts of CD up to 30 equivalents (e.g., 1 eq γ-CD = [NaP_5_W_30_] = 1 mmol·L^−1^) are shown in Figure 4A. The starting CV of the POM showed the three characteristic quasi-reversible redox waves at ca. −0.29, −0.43, and −0.59 V vs. Ag/AgCl on the forward scan wherein the two first redox events involve four electrons [31,32]. Note that the interpretation of the third wave is more complex since protonation can occur. In the presence of the CD, the variation of the redox properties appeared weak, but significant enough to be noticed especially for the first two waves. While almost no significant changes in half-wave potentials could be detected, a substantial decrease of the peak currents was however observed upon addition of the cyclic macromolecule (see Figure 4B). This may be an indication of supramolecular interaction involving the POM which diffuses with a slower rate in the aqueous electrolyte by comparison to the same solution in the absence of CD. The observed decrease in current density occurred in two stages. A first shift was observed from the beginning of the titration to 5 equivalents of CD, followed by a weak continuous decrease until 30 equivalents. Such behavior was observed for both cathodic and anodic peaks. 

**Figure 5:**
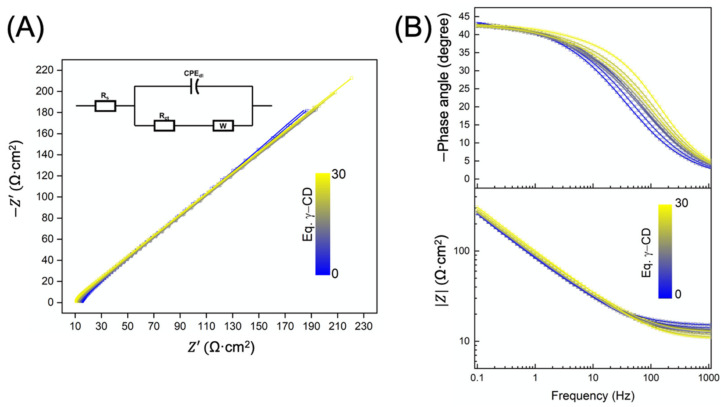
(**A**) Nyquist diagram and (**B**) Bode diagram of the NaP_5_W_30_ anion (1 mmol·L^−1^, glassy carbon working electrode) in the presence of increasing amounts of γ-CD (from 0 to 30 equivalents). The experiments were performed at −0.29 V vs. Ag/AgCl in 50:50 (*v:v*) HCl (0.1 mol·L^−1^):NaCl (0.9 mol·L^−1^) aqueous solution.

To better understand the observed current density decrease and to study the electrochemical reaction mechanism at the surface of the electrode, electrochemical impedance measurements were carried out at −0.29 and −0.43 V vs. Ag/AgCl. The impedance data for the first (Figure 5) and the second (Figure S8) cathodic waves were fitted using the equivalent circuit shown in Figure 5A. 

The proposed mechanisms at −0.29 and −0.43 V vs. Ag/AgCl described by the equivalent circuit are illustrated in Figure S9. The first element of resistance (*R_s_*) characterizes the solution resistance. The reduction of POM (or POM–CD) molecules approaching to the electrode surface, at a defined potential, is described by the charge transfer resistance (*R_ct_*) also known as the faradaic resistance. The constant phase element (CPE*_dl_*) corresponding to a non-ideal capacitor characterizes a double electric layer whose capacitance (*C_dl_*) can be determined using Equation (1) [33].

(1) $$C_{dl}=Q^{\frac{1}{\alpha }}{\left(R_{s}^{-1}+R_{ct}^{-1}\right)}^{\left(\alpha -1\right)/\alpha }$$

with *Q* the specific CPE capacity and *α* (0 < *α* < 1) the CPE fractional exponent distribution (i.e., CPE behaves as an ideal capacitor for *α* = 1). 

Impedance corresponding to the diffusion of POM (or POM–CD) molecules to the electrode surface is modeled by a Warburg element (W), corresponding to a 45° semi-infinite slope in the Nyquist diagram. This diffusion-induced Warburg element is modeled using three parameters: *R_w_*, *τ_w_* and *β* where *R_w_* is the limiting diffusion resistance (related to the concentration and the mobility of ions), *τ_w_* is the diffusion time constant and *β* is an exponent defining the type of Warburg impedance (i.e., *β* < 0.5 for a finite length diffusion, *β >* 0.5 for a finite space diffusion and *β* = 0.5 for a semi-infinite diffusion) [34]. The calculation of the Warburg coefficient (*σ_w_*) is detailed in Supplementary Materials (Section S5.1). The results of EIS fitting for both reduction reactions are gathered in Table 1.

Solution resistance was found to decrease when adding γ-CD to NaP_5_W_30_ solution (e.g., from ca 15 to 10 Ω·cm^2^ for 0 eq. CD to 30 eq. CD respectively, see Table 1 and Table 2) due to the increase of the concentration of the ionic species.

On the other hand, the two-step reductions of NaP_5_W_30_ became faster with the addition of CD in contradiction with cyclic voltammetry results where the reduction peak current densities decreased, implying a lower reaction rate (Figure 4). This may be attributed to the lower concentration of the POM at the surface of the electrode which accelerates, in a certain manner, the reduction reaction. In fact, Komura et al. reported a decrease in the concentration at the electrode surface of the ferrocene derivative complexes while increasing the concentration of β-CD [35]. These observations were attributed to the fact that the γ-CD adsorbs on the electrode surface limiting the access of NaP_5_W_30_. *R_ct_* of the first reduction of NaP_5_W_30_ was lower than the second reduction indicating a faster reaction rate as expected. Moreover, when the concentration of γ-CD exceeded 5 eq., reaction kinetics of both POM reduction became similar due to the possible decrease of NaP_5_W_30_ concentration at the surface. 

The electrode-electrolyte interface (i.e., Nernst diffusion layer) modifications by the addition of CD was evaluated by comparing the evolution of the double layer capacitance. Table 1 and Table 2 show that *C_dl_* values decreased with the addition of CD and the double layer capacitance of the first reduction was lower than the second reduction. These values are linked to the diffusion layer thickness (*d*) since the vacuum permittivity *ε*_0_ is constant, the specific permittivity of POM (or POM–CD) molecules *ε_r_* and the surface area of the electrode *A* are not significantly affected by the addition of γ-CD Equation (2).

(2) $$C_{dl}=\frac{\varepsilon_{0}\varepsilon_{r}A}{d}$$

**Figure 6:**
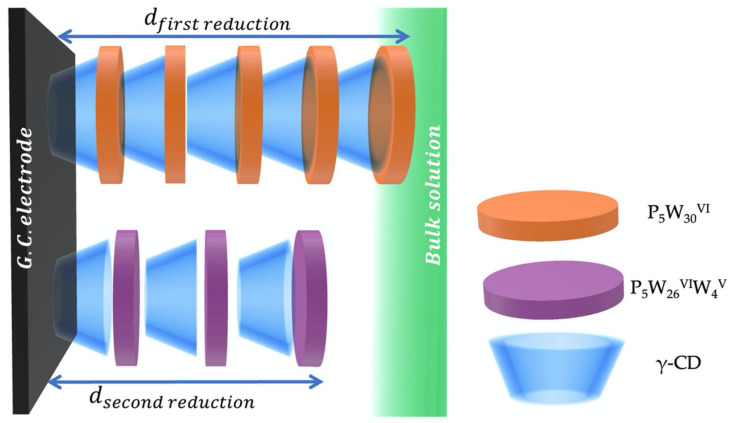
Illustration of the accumulation of POM–CD molecules on the surface of a glassy carbon electrode highlighting the increase in the diffusion layer thickness (*d*) between the first and the second reduction of the POM.

The diffusion layer thickness increase with CD concentration is attributed to the accumulation of the oligosaccharide at the surface of the electrode [35,36]. In addition, *C_dl_* value differences between the first and the second reduction highlight that the diffusion layer was thicker for the first reduction of NaP_5_W_30_. In contrast, α_CPE_ increased from 0.74 to 0.86 and from 0.74 to 0.80 for the first and second reduction reaction, respectively, indicating a mixed adsorption (α → 1) and mass-transfer (α → 0.5) regimes [37]. The adsorption of the POM on the surface of the electrode was slightly slower for P_5_W^VI^_30_–CD (reduction kinetics are limited by the adsorption) since the CPE exponent values for the first reduction reaction were higher than the second one. These observations led us to deduce that the interaction between NaP_5_W_30_ and CD is weaker with the reduced species since the adsorption to the surface is limited by the interaction with the host. Consequently, the “stronger” interaction of POM with γ-CD increases the diffusion layer thickness due to the accumulation of POM–CD on the surface of the electrode as illustrated in Figure 6.

Overall, the decrease of the cathodic peak current densities with the addition of γ-CD is attributed to the accumulation of the oligosaccharide on the surface of the glassy carbon electrode which limits the access of the POM to the electrode. Consequently, less POM is reduced so that the current decreases. However, the Warburg coefficient was calculated to justify this hypothesis. In fact, this latter parameter is directly linked to the concentration of the POM and its diffusion coefficient Equation (3).

(3) $$\sigma_{w}=\frac{RT}{\sqrt{2}An^{2}F^{2}}\left(\frac{1}{\sqrt{D_{Ox}}C_{Ox}}+\frac{1}{\sqrt{D_{Re}}C_{Re}}\right)$$

where *R* is the ideal gas constant, *T* the temperature, *A* the surface area of the electrode, *n* the number of exchanged electrons, *F* the Faraday constant, *D_Ox_*, *D_Re_*, *C_Ox_* and *C_Re_* are the diffusion coefficients and the concentrations of the oxidized and reduced species, respectively. 

Table 1 and Table 2 show that *σ_w_* decreases with the addition of the oligosaccharide. Considering that the diffusion coefficient is slightly modified in this range of concentration of γ-CD (from ca. 2.46 × 10^−10^ to 2.11 × 10^−10^ m^2^·s^−1^) [38], *σ_w_* variations highlight the increase of the concentration in the bulk according to Equation (3). These results confirm our hypothesis since the decrease of NaP_5_W_30_ concentration at the electrode–electrolyte interface led to higher concentration of the POM in the bulk (given that the total concentration of NaP_5_W_30_ is constant), which lower the Warburg coefficient values. 

## 3. Materials and Methods

### 3.1. Reagents

All reagents were purchased from commercial sources and used without further purification. K_14_Na[P_5_W_30_O_110_]•32H_2_O was prepared according to the literature [39]. Fourier transform infra-red (FT-IR) spectra were recorded on a 6700 FT-IR Nicolet spectrophotometer (Madison, WI, USA), using diamond ATR technique. The spectra were recorded on nondilute samples and ATR correction was applied. Elemental analyzes of C and H were carried out by BIOCIS analytical facility (Châtenay-Malabry, France). Quantitative elemental analyzes of Na, K, P, and W were carried out by inductively coupled plasma optical emission spectroscopy (ICP-OES) with an Agilent 720 Series (Santa Clara, CA, USA) with axially-viewed plasma. Energy-dispersive X-ray spectroscopy (EDS) measurements were performed using a SEM-FEG (Scanning Electron Microscope enhanced by field emission gun) equipment (JSM 7001-F, Jeol, Tokyo, Japan). The measures were acquired with a SDD XMax 50 mm^2^ detector and the Aztec (Oxford) system working at 15 kV and 10 mm distance. The quantification was realized with the standard library provided by the constructor using Lα lines. Water and organic contents were determined by thermal gravimetric analysis (TGA) with a Mettler Toledo TGA/DSC 1, STAR^e^ System apparatus (Giessen, Hesse, Germany) under oxygen flow (50 mL·min^−1^) at a heating rate of 5 °C·min^−1^ up to 700 °C. 

### 3.2. NMR Studies

All solution NMR spectra were measured in D_2_O at 28 °C. ^1^H and ^31^P NMR spectra were obtained on a Bruker Avance 400 spectrometer (Billerica, MA, USA) at Larmor frequencies 400.1 and 162.0 MHz respectively, using 5 mm standard NMR tubes. The ^1^H spectra were recorded with one pulse sequence at 30° flip angle (pulse duration 2.4 µs), using 1 s recycle delay, 1.6 s acquisition time, and 16 number of scans. The ^31^P NMR spectra were run with 7.7 µs pulse duration (45° flip angle), 15 s recycle delay, 1 s acquisition time, and an accumulation of 32 transients. Chemical shifts are reported relative to TMS for ^1^H, and 85% H_3_PO_4_ for ^31^P.

### 3.3. Electrochemistry

Purified water was used throughout. It was obtained by passing water through a RiOs 8 unit followed by a Millipore-Q Academic purification set (Merck KGaA, Darmstadt, Germany). All reagents were of high-purity grade and were used as purchased without further purification. Cyclic voltammetry (CV) and electrochemical impedance spectroscopy (EIS) experiments were carried out with a Metrohm Autolab PGSTAT12 potentiostat/galvanostat (Villebon Courtaboeuf, France) associated with a GPES electrochemical analysis system (EcoChemie). Measurements were performed at room temperature in a conventional single compartment three-electrode cell. A glassy carbon electrode with a diameter of 3 mm was used as the working electrode. The auxiliary electrode was a Pt plate placed within a fritted-glass isolation chamber and potentials were quoted against a silver chloride electrode (*E*°_Ag/AgCl sat. KCl_ = 197 mV vs. SHE). EIS measurements were carried out over a frequency ranging from 1 kHz to 100 mHz with a 10 mV amplitude sinusoidal voltage. The solutions were de-aerated thoroughly for at least 30 min with pure argon and kept under a positive pressure of this gas during the experiments.

### 3.4. Single-Crystal X-ray Diffraction Analysis

Intensity data collections were carried out at T = 200(2) K with a Bruker D8 VENTURE diffractometer (Karlsruhe, Germany) equipped with a PHOTON 100 CMOS bidimensional detector using a high brilliance IμS microfocus X-ray Mo Kα monochromatized radiation (λ = 0.71073 Å). Crystals were glued in paratone oil to prevent any loss of crystallization water. Data reduction was accomplished using SAINT V7.53a. The substantial redundancy in data allowed a semi-empirical absorption correction (SADABS V2.10) to be applied, on the basis of multiple measurements of equivalent reflections. Using Olex2 [40], the structure was solved with the ShelXT [41] structure solution program using intrinsic phasing and refined with the ShelXL [42] refinement package using least squares minimization. Tungsten atoms were initially located by direct methods. The remaining nonhydrogen atoms (P, K, Na, O and C) were located from Fourier differences and were refined with anisotropic thermal parameters. Positions of the hydrogen atoms belonging to the γ-CDs were calculated and refined isotropically using the gliding mode. In the compound, free water molecules (35.25 H_2_O per NaP_5_W_30_) and counterions (4.88 K^+^ and 5 Na^+^ per NaP_5_W_30_) located inside the voids are disordered. Thereby, the contribution of solvent-electron density was removed using the SQUEEZE routine in PLATON, producing a set of solvent-free diffraction intensities. Crystallographic data for single-crystal X-ray diffraction studies are summarized in Table S1 in the Supplementary Materials. CCDC number: 2080086.

### 3.5. Synthesis of K_6_Na_8_{[NaP_5_W_30_O_110_]•(C_48_H_80_O_40_)}•23H_2_O (**NaP_5_W_30_•1CD**)

K_14_Na[P_5_W_30_O_110_]•32H_2_O (0.3 g, 0.038 mmol) was dissolved in 10 mL aqueous solution of NaCl (0.2 mol·L^−1^) and γ-CD (0.05 g, 0.038 mmol) was added. The resulting limpid solution was stirred at room temperature for 30 min. The solution was then allowed to stand at room temperature for crystallization in air where rod/stick-like colorless crystals of **NaP_5_W_30_•1CD** suitable for single crystal X-ray diffraction appeared within 7 days. They were isolated by filtration and washed with cold water. Yield 0.23 g, 71%. FT-IR spectrum is given in Figure S1. Elemental analysis for K_6_Na_8_{[NaP_5_W_30_O_110_](C_48_H_80_O_40_)}•23H_2_O (Mw = 9582.97 g·mol^−1^) Calc. (found): H 1.33 (0.97); C 6.02 (6.03); Na 2.16 (2.28); K 2.45 (2.16); P 1.62 (1.54); W 57.55 (55.5). TGA (Figure S2) showed a weight loss of 4.4% in the 20–200 °C temperature range corresponding to the hydration water (calculated 4.4%) and a weight loss of 14.0% in the 200–700 °C range assigned to the loss of 1 CD (calculated 13.9%).

## 4. Conclusions

A new supramolecular association between γ-CD and the Preyssler-type anion [NaP_5_W_30_O_110_]^14−^ consisting of a 1:1 POM–CD adduct has been isolated and characterized by single-crystal X-ray diffraction. Structural analysis revealed that γ-CD interacts with its primary face as usually observed with bulky POMs. The study of the behavior of the cyclodextrin in solution by the ^1^H NMR technique confirmed such an interaction with H6 protons suggesting that a similar supramolecular 1:1 arrangement persists in solution. Although weak, the recognition process is most probably dominated by the chaotropic effect of this highly charged anionic species. The decrease of the reduction peaks’ current densities was studied by electrochemical impedance spectroscopy, evidencing the reduced accessibility of the POM at the surface of the electrode due to the adsorption of the γ-CD. 

## Figures and Tables

**Table 1:** Electrochemical parameters obtained from the best fit of impedance data for POM and POM–CD reduction reaction at the first cathodic peak (−0.29 V vs. Ag/AgCl). Data obtained from EIS fitting using ZView software is shown in Table S2.

	Eq. γ-CD	*R_s_* (Ω·cm^2^)	*C_dl_* (µF·cm^−2^)	*R_ct_* (Ω·cm^2^)	*σ_w_* (Ω·cm^2^)
**First cathodic peak**	0	14.8	384.1	24.2	6.7
	0.5	13.7	314.5	18.3	5.0
	1	12.1	289.0	18.6	2.4
	1.5	12.6	250.8	17.0	2.1
	2	13.2	251.9	15.2	1.9
	3	12.9	240.8	14.7	1.9
	4	12.2	231.0	14.0	1.8
	5	11.6	222.0	13.5	1.7
	10	10.9	208.9	13.2	1.7
	20	10.6	203.8	14.1	1.9
	30	10.5	197.5	14.6	2.0

**Table 2:** Electrochemical parameters obtained from the best fit of impedance data for POM and POM–CD reduction reaction at the second cathodic peak (−0.43 V vs. Ag/AgCl). Data obtained from EIS fitting using ZView software is shown in Table S2.

	Eq. γ-CD	*R_s_* (Ω·cm^2^)	*C_dl_* (µF·cm^−2^)	*R_ct_* (Ω·cm^2^)	*σ_w_* (Ω·cm^2^)
**Second cathodic peak**	0	14.7	503.4	30.5	4.4
	0.5	13.6	442.9	26.3	3.4
	1	12.0	391.8	20.8	2.7
	1.5	12.6	381.0	21.2	2.6
	2	13.1	370.9	20.0	2.5
	3	12.8	374.1	21.9	2.7
	4	12.2	357.2	20.7	2.7
	5	11.5	333.8	18.5	2.3
	10	10.8	306.1	16.0	2.0
	20	10.4	286.9	15.7	2.0
	30	10.2	267.5	14.8	1.9

## Data Availability

Not applicable.

## Supplementary Materials

The following are available online. Figure S1: FT-IR spectrum of **NaP_5_W_30_•CD**, K_14_Na[P_5_W_30_O_110_]•32H_2_O (NaP_5_W_30_) and γ-cyclodextrin (CD). Figure S2: Thermogravimetric curve of freshly prepared **Na****P_5_W_30_•CD.** Figure S3: Structural representation of the **{NaP_5_W_30_}•1CD** arrangement showing the involvement of the potassium and sodium cations within the POM•••γ-CD interactions. Figure S4: Representation of the **{NaP_5_W_30_}•1CD** structure showing chains of the POM and of the CD running along the *a* and *c* axes. Figure S5: ^1^H NMR spectra of γ-CD aqueous solution containing variable amounts of K_14_Na[P_5_W_30_O_110_]. Figure S6: ^1^H NMR titration curve of γ-CD with the POM. Figure S7: ^31^P NMR spectra of K_14_Na[P_5_W_30_O_110_] and **{NaP_5_W_30_}•1CD** aqueous solutions. Figure S8: Nyquist and Bode diagrams of the **NaP_5_W_30_** anion in the presence of increasing amounts of γ-CD. Figure S9: Correspondence of equivalent electric circuit to the proposed schematic mechanism of the electrochemical processes at −0.29 and −0.43 V vs. Ag/AgCl. Table S1: Crystal data for **NaP_5_W_30_•1CD**. Table S2: Electrochemical parameters calculated using ZView software for POM and POM–CD reduction reactions at −0.29 and −0.43 V vs. Ag/AgCl.

## Sample Availability

Samples of the compounds are available from the authors.
