# Supplementary material for: Supramolecular Association between γ-Cyclodextrin and Preyssler-Type Polyoxotungstate
Source: Molecules. 2021 Aug 24;26(17):5126. doi: 10.3390/molecules26175126 (PMC8434062; doi:10.3390/molecules26175126)
Supplement: Supplementary file 1 [file molecules-26-05126-s001.zip › molecules-1326508-supplementary.pdf]

Supramolecular Association Between γ–Cyclodextrin and Preyssler-Type Polyoxotungstate

Nathalie Leclerc,[a]* Mohamed Haouas,[a] Clément Falaise,[a] Serge Al Bacha,[a,b] Loïc Assaud[b] and Emmanuel Cadot[a]

[a] *Institut Lavoisier de Versailles, UMR 8180 CNRS, UVSQ, Université Paris-Saclay, 78035 Versailles Cedex, France.*

[b] *Institut de Chimie Moléculaire et des Matériaux d'Orsay (ICMMO) – ERIEE, UMR 8182 CNRS, Université Paris-Saclay, 91400, Orsay, France.*

Supporting information

Table of Contents

1. FT-IR Spectra S2

2. Thermogravimetric analysis S3

3. Single crystal X-ray diffraction S4

3.1. Selected crystallographic parameters of the single-crystal X-ray diffraction structural analysis S4

3.2. Structural views of POM•1CD arrangement S5

4. NMR data S7

4.1. ^{1}H NMR titration S7

4.2. ^{31}P NMR S8

5. Electrochemical impedance spectroscopy S9

5.1. Warburg coefficient S9

5.2. EIS data S9

1. FT-IR Spectra

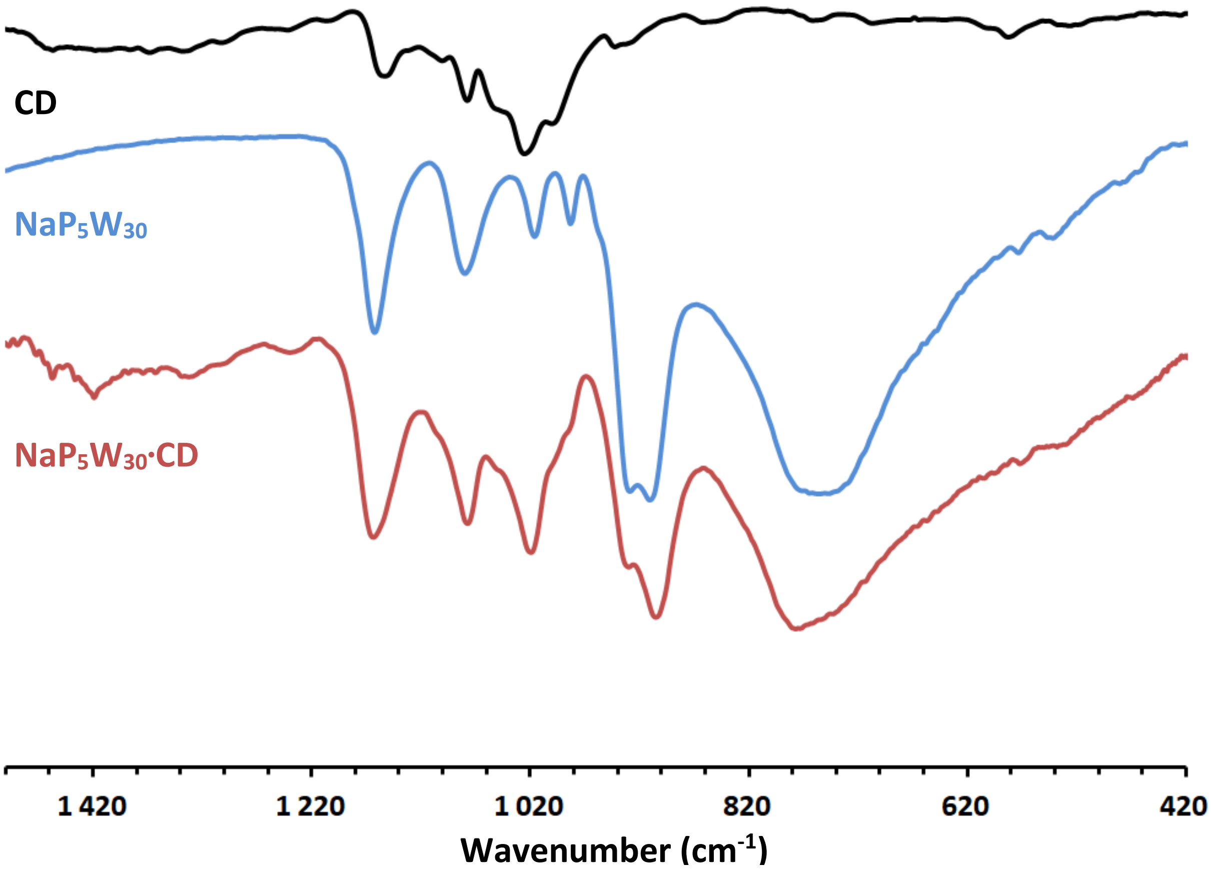

Figure S1. FT-IR spectrum of compound **NaP_5W_{30}•CD**, in comparison with the spectra of the precursor $K_{14}Na[P_5W_{30}O_{110}]$•$32H_2O$ (NaP_5W_{30}) and of the γ-cyclodextrin (CD). From NaP_5W_{30}•CD, the ratio of the bands of CD compared to that of the POM clearly increases (see the 1020 cm^{-1} band).

2. Thermogravimetric analysis

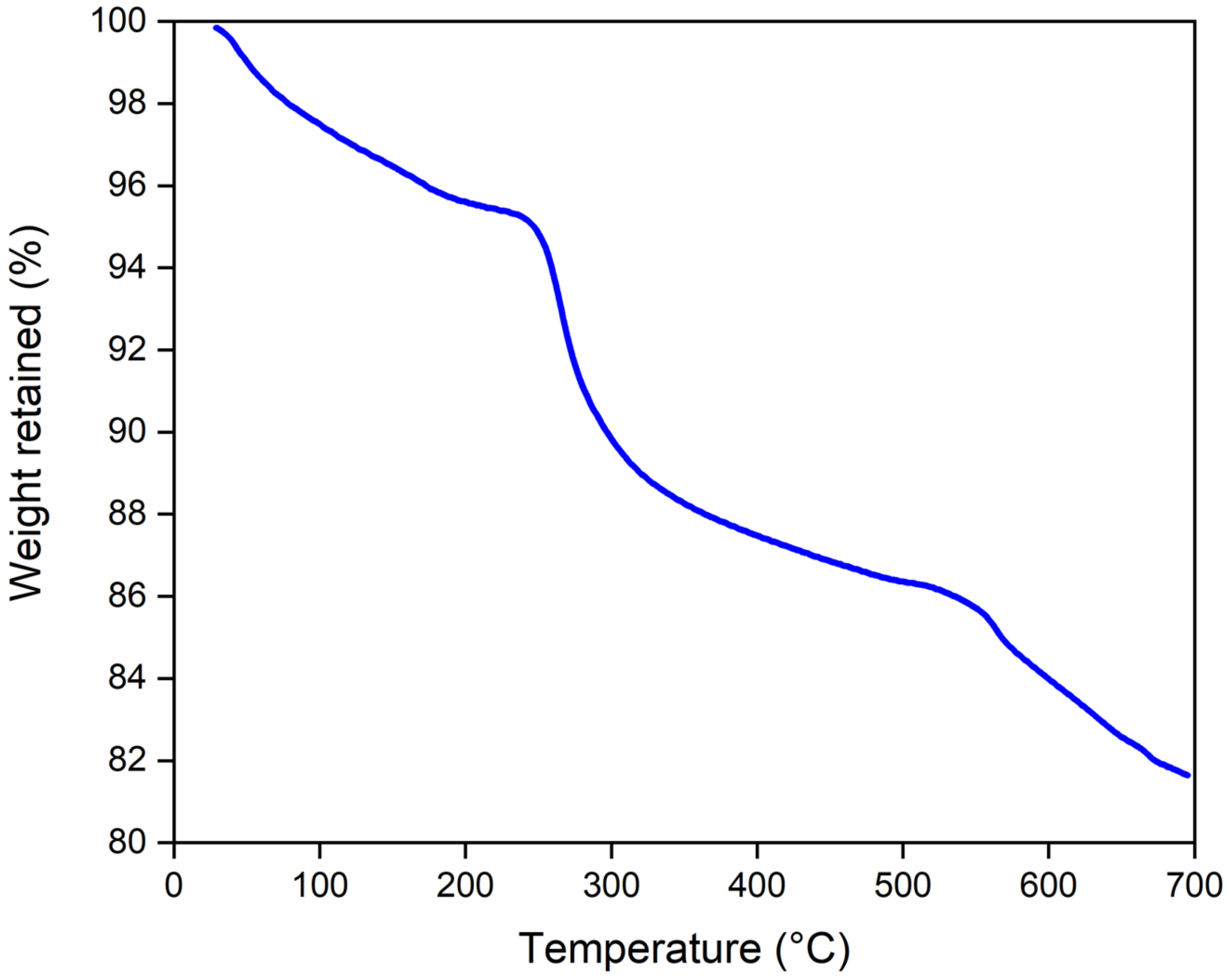

Figure S2. Thermogravimetric curve of freshly prepared **NaP_5W_{30}•CD**.

3. Single crystal X-ray diffraction

3.1. Selected crystallographic parameters of the single-crystal X-ray diffraction structural analysis

Table S1. Crystal data for NaP_5W_{30}•1CD.

Identification code	NaP_5W_{30}•1CD
**Empirical formula**	$C_{48}H_{55}K_{4.88}Na_6O_{185.25}P_5W_{30}$
**Formula weight**	9594.82
**Temperature/K**	200
**Crystal system**	Monoclinic
**Space group**	$P2_1$
***a*/Å**	40.522
***b*/Å**	20.995
***c*/Å**	25.108
***α*/ °**	90
***β*/ °**	98.55
***γ*/ °**	90
**Volume/Å^3**	21124.3
**Z**	4
**ρ_{calc}g/cm^3**	3.017
**μ/mm^{-1}**	16.521
**F(000)**	17114.0
**Crystal size/mm^3**	0.15 × 0.12 × 0.1
**Radiation**	MoKα (λ = 0.71073)
**2Θ range for data collection/°**	4.01 to 56.824
**Index ranges**	$-54 \leq h \leq 54$<br>$-28 \leq k \leq 28$<br>$-33 \leq l \leq 33$
**Reflections collected**	390618
**Independent reflections**	105407<br>R_{int} = 0.0726<br>R_{sigma} = 0.0603
**Data/restraints/parameters**	105407/62/2345
**Goodness-of-fit on F^2**	1.049
**Final R indexes [I>=2σ (I)]**	R_1 = 0.0490<br>wR_2 = 0.1182
**Final R indexes [all data]**	R_1 = 0.0546<br>wR_2 = 0.1228
**Largest diff. peak/hole / e Å^{-3}**	3.95/-3.76
**Flack parameter**	0.111(9)

3.2. Structural views of POM•1CD arrangement

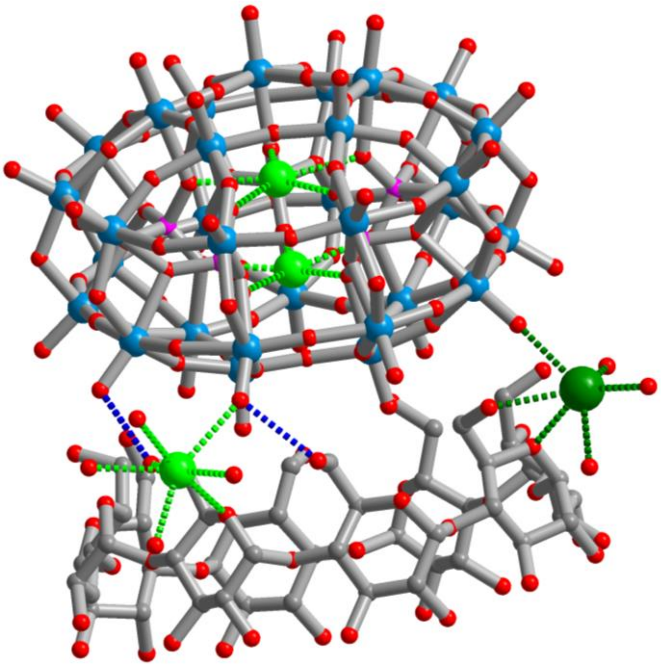

Figure S3. Structural representation of the **POM•1CD** arrangement showing the involvement of the potassium cations (dark-green spheres) and sodium cations (light-green spheres) within the POM•••γ-CD interactions in addition to the hydrogen bonds (dotted blue sticks).

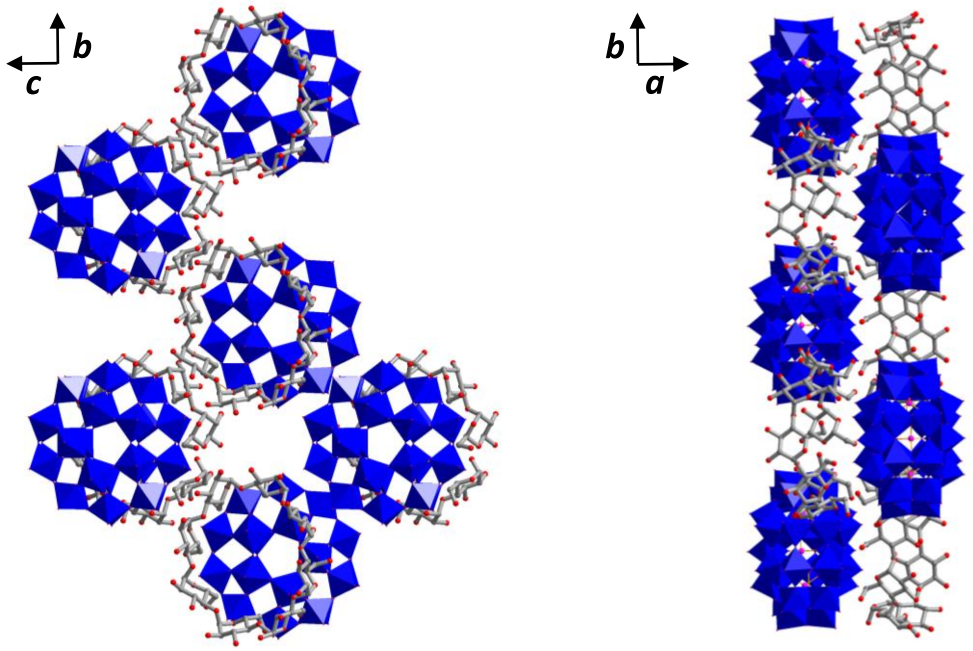

Figure S4. Representation of the **$\{NaP_5W_{30}\}$•1CD** structure showing chains of the POM and of the CD running along the *a* and *c* axes. The supramolecular host-guest assembly are alternately stacked through short and long intermolecular interactions involving the primary face of the γ-CD. These chains are mutually connected through the sodium/potassium counterions to give the monoclinic cell. Color code: blue polyhedral represent the W-O framework of the $\{NaP_5W_{30}\}^{14-}$ POM; the cyclodextrin are represented by grey sticks.

4. NMR data

4.1.^{1}H NMR titration

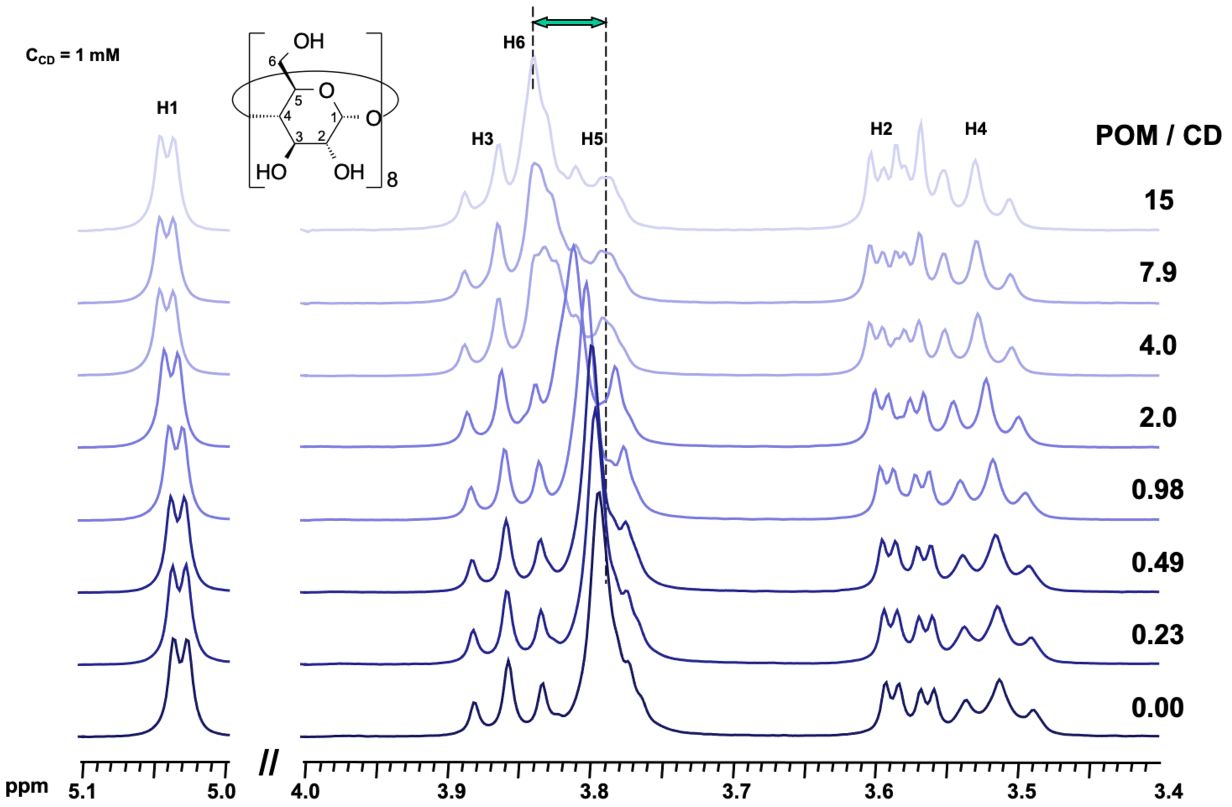

Figure S5. ^{1}H NMR spectra of γ-CD aqueous solution (3 mM) containing variable amounts of $K_{14}Na[P_5W_{30}O_{110}]$, showing effect on signal H_6 as a result of complex formation on the primary rim of the CD.

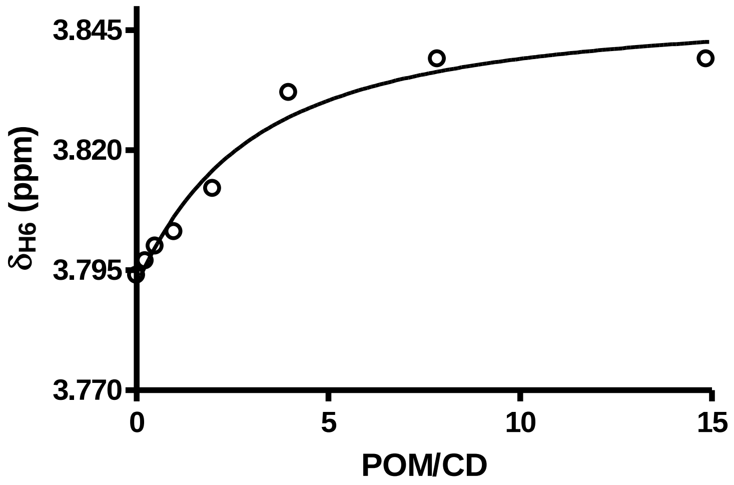

Figure S6. ^{1}H NMR titration curve of γ-CD with the POM. A plot of the chemical shifts of the higher field H6 proton of the γ-CD as a function of POM/CD molar ratio was used to estimate the association constant K (430 ± 60 M^{-1}) for the 1:1 complex.

4.2. ^{31}P NMR

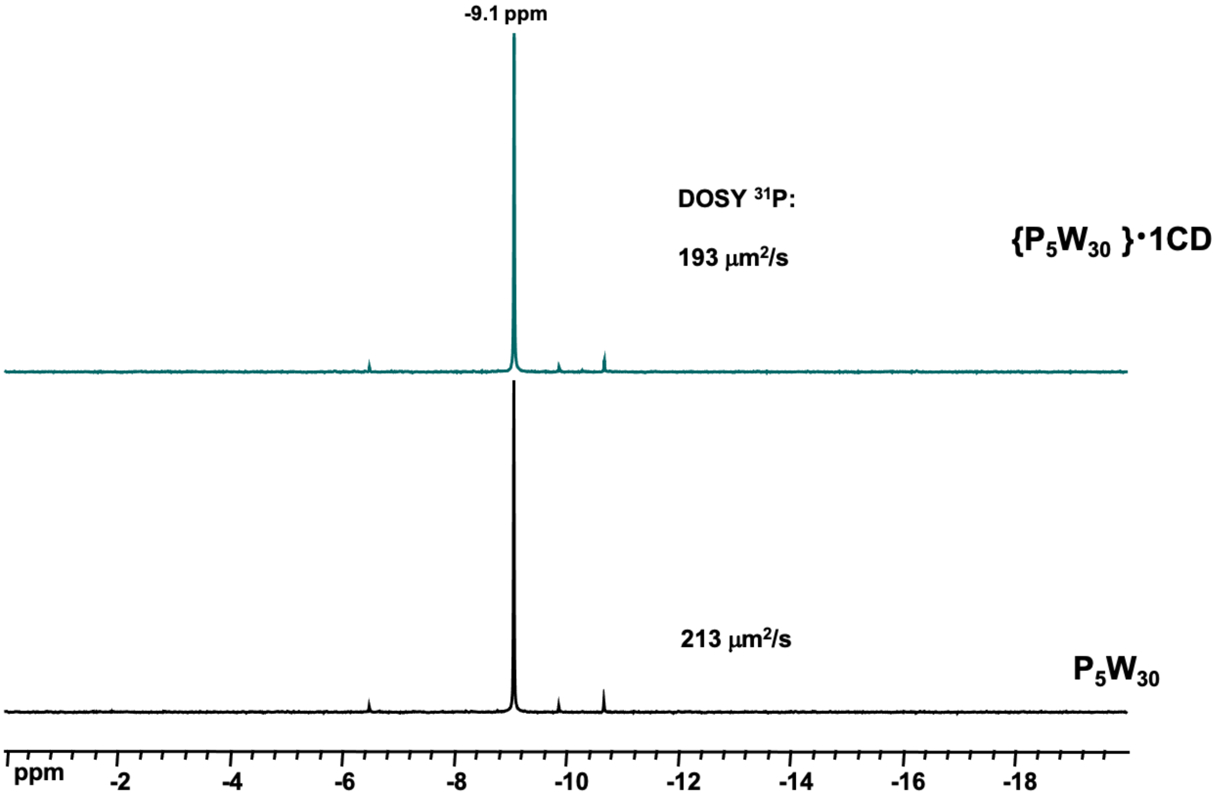

Figure S7. ^{31}P NMR spectra of $K_{14}Na[P_5W_{30}O_{110}]$ and **{NaP$_5$W$_{30}$}•1CD** aqueous solutions showing no effect on the POM signal.

5. Electrochemical impedance spectroscopy

5.1.Warburg coefficient

Short Warburg impedance $\mathbf{Z_w}$ is defined as:

$$Z_w = \frac{\sigma_w}{(i\omega)^{\beta}} \tanh\left[(i\tau_w\omega)^{\beta}\right] \quad \text{Eq. S1}$$

With ZView software, the same impedance is calculated using:

$$Z_w = \frac{R_w}{(i\tau_w\omega)^{\beta}} \tanh\left[(i\tau_w\omega)^{\beta}\right] \quad \text{Eq. S2}$$

By comparing Eq. S1 to Eq. S2, Warburg coefficient $\boldsymbol{\sigma_w}$ is deduced as follows:

$$\sigma_w = \frac{R_w}{(\tau_w)^{\beta}} \quad \text{Eq. S3}$$

Given the fact that *ß* was also fitted for EIS data analysis, the comparison of $\mathbf{R_w}$ and $\boldsymbol{\tau_w}$ values cannot be justified.

5.2.EIS data

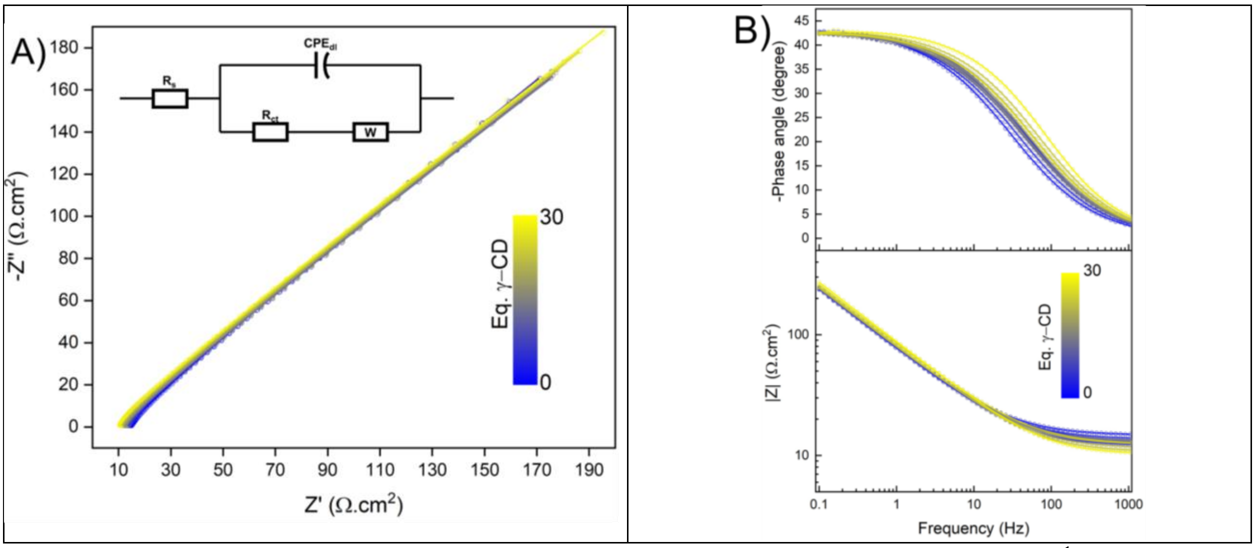

Figure S8. A) Nyquist diagram and B) Bode diagram of the $\mathbf{NaP_5W_{30}}$ anion (1 mmol.L^{-1}, glassy carbon working electrode) in the presence of increasing amounts of γ-CD (from 0 to 30 equivalents). The experiments have been performed at -0.43 V *vs* Ag/AgCl in 50:50 (v:v) HCl (0.1 mol.L^{-1}):NaCl (0.9 mol.L^{-1}) aqueous solution.

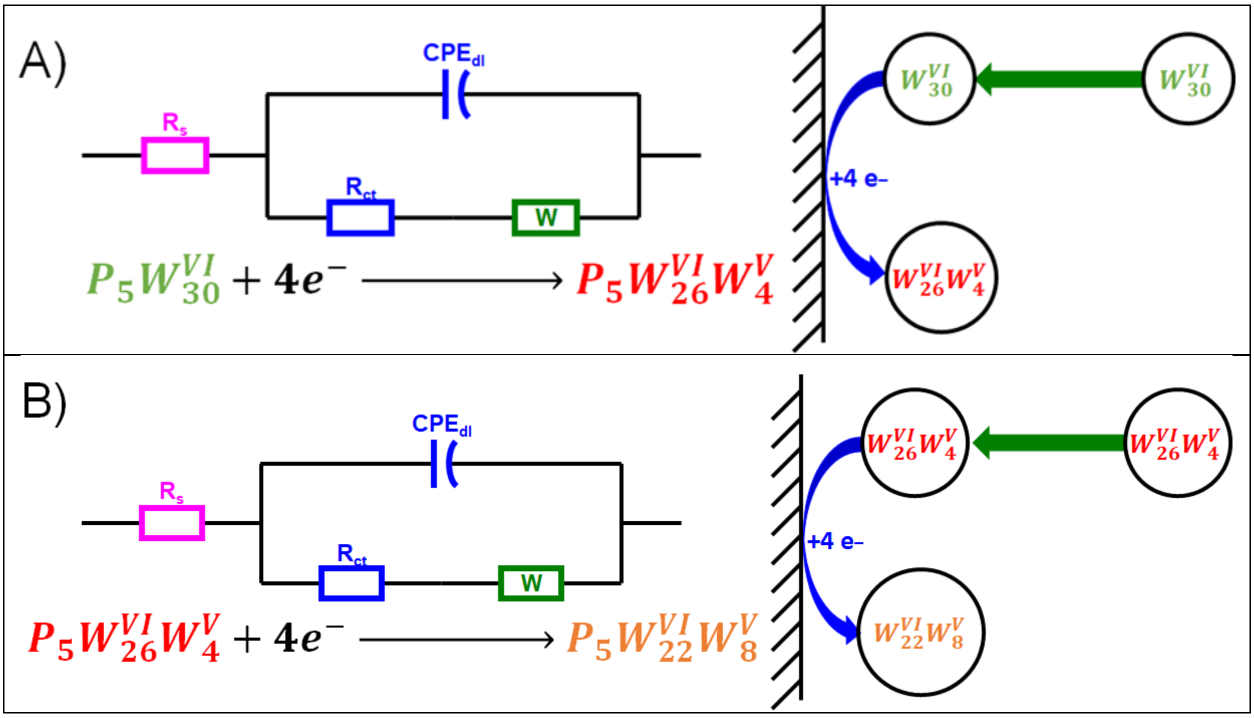

Figure S9. Correspondence of equivalent electric circuit to the proposed schematic mechanism of the electrochemical processes at A) -0.29 and B) -0.43V *vs* Ag/AgCl. (For better interpretation, the reader is referred to the online version).

Table S2. Electrochemical parameters calculated using ZView software for POM and POM–CD reduction reactions at A) -0.29 and B) -0.43 V *vs* Ag/AgCl.

A)	Eq. γ–CD	R_s (Ω.cm²)	Q (mF.cm⁻².s⁻ᵅ)	α	R_{ct} (Ω.cm²)	R_w (Ω.cm².s⁻ᵝ)	τ_w (s)	ß
First cathodic peak	**0**	14.8	1.67	0.740	24.2	921	16.5	0.457
	**0.5**	13.7	1.32	0.761	18.3	883	16.8	0.460
	**1**	12.1	1.19	0.771	18.6	1844	79.7	0.468
	**1.5**	12.6	0.88	0.801	17.0	1871	81.8	0.475
	**2**	13.2	0.95	0.790	15.2	1831	81.0	0.472
	**3**	12.9	0.87	0.799	14.7	1753	72.0	0.473
	**4**	12.2	0.80	0.809	14.0	1786	75.8	0.476
	**5**	11.6	0.73	0.820	13.5	1794	75.5	0.478
	**10**	10.9	0.61	0.841	13.2	1788	67.7	0.482
	**20**	10.6	0.55	0.851	14.1	1793	65.4	0.483
	**30**	10.5	0.51	0.859	14.6	1833	59.6	0.486

B)	Eq. γ–CD	R_s (Ω.cm²)	Q (mF.cm⁻².s⁻ᵅ)	α	R_{ct} (Ω.cm²)	R_w (Ω.cm².s⁻ᵝ)	τ_w (s)	ß
Second cathodic peak	**0**	14.7	2.03	0.737	30.5	1679	68.3	0.457
	**0.5**	13.6	1.84	0.742	26.3	1809	87.9	0.456
	**1**	12.0	1.59	0.759	20.8	1740	86.8	0.455
	**1.5**	12.6	1.57	0.756	21.2	1939	102.9	0.456
	**2**	13.1	1.56	0.754	20.0	1845	97.5	0.455
	**3**	12.8	1.60	0.749	21.9	1877	98.3	0.454
	**4**	12.2	1.53	0.753	20.7	1807	89.1	0.456
	**5**	11.5	1.41	0.762	18.5	1825	91.5	0.457
	**10**	10.8	1.24	0.775	16.0	1857	94.0	0.460
	**20**	10.4	1.11	0.786	15.7	1810	82.2	0.463
	**30**	10.2	0.97	0.800	14.8	1787	78.9	0.466
